# A randomized controlled trial testing a social network intervention to promote physical activity among adolescents

**DOI:** 10.1186/s12889-018-5451-4

**Published:** 2018-04-23

**Authors:** Thabo J. van Woudenberg, Kirsten E. Bevelander, William J. Burk, Crystal R. Smit, Laura Buijs, Moniek Buijzen

**Affiliations:** Behavioural Science Institute, Radboud University, Nijmegen, The Netherlands

**Keywords:** Social network intervention, Physical activity, Accelerometer, Adolescents, Smartphones

## Abstract

**Background:**

The current study examined the effectiveness of a social network intervention to promote physical activity among adolescents. Social network interventions utilize peer influence to change behavior by identifying the most influential individuals within social networks (i.e., influence agents), and training them to promote the target behavior.

**Method:**

A total of 190 adolescents (46.32% boys; *M* age = 12.17, age range: 11–14 years) were randomly allocated to either the intervention or control condition. In the intervention condition, the most influential adolescents (based on peer nominations of classmates) in each classroom were trained to promote physical activity among their classmates. Participants received a research smartphone to complete questionnaires and an accelerometer to measure physical activity (steps per day) at baseline, and during the intervention one month later.

**Results:**

A multilevel model tested the effectiveness of the intervention, controlling for clustering of data within participants and days. No intervention effect was observed, *b* = .04, *SE* = .10, *p* = .66.

**Conclusion:**

This was one of the first studies to test whether physical activity in adolescents could be promoted via influence agents, and the first social network intervention to use smartphones to do so. Important lessons and implications are discussed concerning the selection criterion of the influence agents, the use of smartphones in social network intervention, and the rigorous analyses used to control for confounding factors.

**Trial registration:**

Dutch Trial Registry (NTR): NTR6173. Registered 5 October 2016 Study procedures were approved by the Ethics Committee of the Radboud University (ECSW2014–100614-222).

## Background

Physical activity in childhood and adolescence is linked to numerous health benefits, such as lower cholesterol, blood pressure and BMI [1]. People who are more physically active at a young age are also more active adults [2]. Unfortunately, young people are not physically active enough and physical activity declines with age [3, 4]. Nowadays, adolescents are even less physically active compared to previous generations [5]. According to the World Health Organization (2011), adolescents should accumulate at least 60 min of moderate-to-vigorous physical activity (MVPA) every day. Yet, a worldwide majority (80%) of adolescents, aged 13 to 15 year old, are not meeting these guidelines [6–8]. In the United States, for example, 93% of adolescents (12- to 15-year-olds) do not meet the recommended amount of physical activity [9] and in the Netherlands (the country of the current study), 72% of the adolescents (12- to 17-year-olds) do not adhere to the norm of 60 min of MVPA per day [10].

Physical activity of adolescents is found to be influenced by peers [11, 12]. For example, studies have shown that adolescents are more active when they are together with peers [13] and that adolescents are more often friends with others who are similar in terms of physical activity [14]. In addition, some studies have used a social network framework to predict physical activity in youth. For example, a study by De La Haye, Robins, Mohr, and Wilson [15] showed that adolescents (12- to 14-year-olds) selected friends based on the amount of self-reported MVPA, but also influenced the amount of physical activity of their friends. Similarly, Simpkins, Schaefer, Price and Vest [16] found evidence for these so-called selection and influence effects, based on self-reported physical activity in adolescents (*M* age = 15.97). Gesell, Tesdahl and Ruchman [17] observed only friendship selection effects in children and adolescents (5- to 12-year-olds), based on physical activity measured by accelerometer. All together, these studies show the relationship between adolescents’ physical activity and the physical activity of friends and peers, and that it is plausible that physical activity can be influenced by their social network.

A social network framework can be used to design interventions for behaviors in which peer influence plays a crucial role [18]. Social network interventions typically identify a small number of individuals within social networks, so-called *influence agents*, and train these agents to promote specific behaviors within their networks. There are a number of ways in which influence agents are selected [19]. Usually, influence agents are selected by choosing participants that are nominated most frequently by all members of the social network on one or more sociometric questions (e.g., regarding who they respect, want to be like or who are their friends; see [20, 21]). Once the influence agents have been selected, they are approached and trained to promote the desired behavior in their network for intervention purposes. Previous research has shown promising results that influence agents can stimulate healthy behaviors, such as a healthy eating [22] and water consumption [23], or discourage unhealthy behaviors, such as smoking [20, 21] and substance use [24].

Despite the promising approach of using influence agents to promote health behavior, only two studies have tested a social network intervention to promote physical activity in adolescents [25, 26]. Both studies were based on the ASSIST study framework [20], in which influence agents are trained to promote or discourage behavior among their peers. Bell et al. [25] selected the most nominated adolescents as influence agents and trained them in a two-day training session to promote healthy eating and physical activity at the same time. After a 10-week intervention period, no behavioral differences were observed between the control and intervention conditions. The authors suggested that it was too complicated for the influence agents to promote both health behaviors at the same time. The second study [26] focused solely on physical activity of adolescent females. The most nominated female adolescents in each classroom were selected as influence agents. The influence agents received a three-day training program about physical activity and interpersonal communication skills. After the training, the influence agents were asked to informally diffuse messages about physical activity for a period of 10 weeks. Preliminary results suggest that this intervention was successful [27]. That is, adolescent girls decreased less in MVPA compared to the control condition. These mixed findings show that more research is needed on social network interventions that promote physical activity.

### Current study

This study extends research on social network interventions aimed at promoting adolescents’ physical activity by (a) using a different selection criterion to determine the influence agents, and (b) training the influence agents via smartphones.

First, this study used closeness centrality as the selection criterion to determine the influence agents. In previous social network interventions, influence agents have been selected by identifying participants in the network who received the most nominations on one or more sociometric questions. This selection criterion is referred to as *indegree centrality*. In most cases, the participants with the highest indegree centrality are the most popular individuals within a classroom. However, this might impair the effectiveness of the intervention, because popularity could be a detrimental characteristic of influence agents [28]. For example, Valente argued that popular adolescents often depend on the social norms of the network to remain popular, and therefore may be reluctant to change their behavior or perform the role of an influence agent. As a solution, Borgatti [29, 30] reasoned that when an intervention aims to promote health behavior, one should select the influence agents based on *closeness centrality*. Based on this criterion, the influence agents are those in a classroom who are closely connected to all other classmates. More specifically, closeness refers to how many relationship ties are needed to link an individual to all others in a social network. Closeness centrality is calculated by taking the sum of the length of the shortest paths between each participant and all the classmates. People who have a small average path length, need fewer intermediaries to reach all members of a network. Therefore, it takes less time (i.e., fewer interactions) for the intervention message to reach the entire classroom [30]. For this reason, the current intervention selected the influence agents based on closeness centrality.

Second, this study used smartphones to train the influence agents. Typically, influence agents are trained using repeated face-to-face meetings with trained experts. Delivering the training via smartphones increased the feasibility of social network interventions because it is a low-cost and less time-consuming method [31]. For example, the influence agents can be trained at any location and time without having to miss part of their school curriculum. In addition, the use of smartphones fits adolescents’ lifestyle and the training of influence agents can be done covertly without raising suspicion of their peers, because they do not have to leave the classroom to attend the training.

The aim of this study was to test the effectiveness of a social network intervention that promotes physical activity in adolescents, based on these two extensions. We hypothesized that adolescents who are exposed to the social network intervention would be more physically active than adolescents who are not exposed to the social network intervention.

## Method

### Design

The study used a clustered randomized control trial design of two groups. Participating classes were randomly allocated to the intervention condition (social network intervention) or the control condition (no intervention). The study was registered a priori in the Dutch Trial Registry (NTR): TR6173^1^ and the procedures were approved by the Ethics Committee of the Radboud University (ECSW2014–100614-222).

**Fig. 1:**
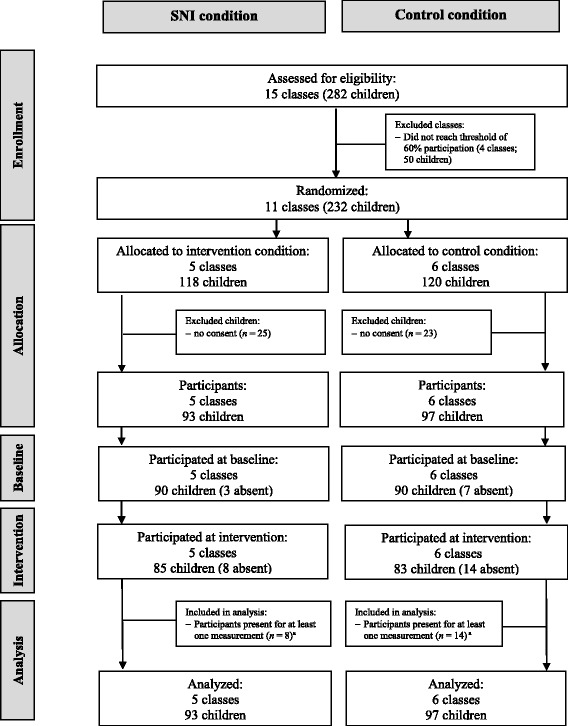
CONSORT flow diagram of participants. ^a^Because of imputation of the data, participants with only one week of data could be included in the analysis

A priori sample size calculation was performed by using G*Power 3.1 [32]. For the calculation, the observed effect size in the study by Smit et al. [23] was used (η^2^ = .07) and converted to Cohen’s *F* (*f* = 0.25). The calculation showed that 130 participants were needed for a MANCOVA: repeated measures within-between interaction with two groups and two measurements (power = 0.80, *p* = 0.05). A larger number of participants were recruited due to the strictness of the inclusion criteria (i.e., active parental consent, minimum of 60% classroom participation) and to account for attrition (see Fig. 1).

### Participants and procedure

In total, 326 first year pupils from 15 secondary classes of a Dutch secondary school (region Venlo) were approached in September – October 2016 to participate in the study via their school. Parents or legal guardians received an information letter about the project with the corresponding consent form. Active parental consent was obtained for 219 students. We limited participation to classrooms in which at least 60% of students provided consent. This was done to ensure a reliable assessment of the social networks [33]. Four classes did not reach this threshold and were excluded from the study. After exclusion, the sample consisted of 11 classes with 190 participants (46% male) ranging from 11 to 14 years old (*M* = 12.17 years, *SD* = 0.50). The level of education of the classes varied, ranging from the lowest education level (“VMBO-kader”, vocational training) to a moderate-high level (“HAVO/VWO”, theoretical training). Five classes (*n* = 93) were assigned to the intervention condition and six classes (*n* = 97) to the control condition (see Fig. 1). All participants signed assent before receiving the materials.

The baseline measures were administered over a seven day period (November 2016) followed by a seven day intervention one month later (December 2016). At the start of the baseline measurement, the participants received instructions about the project and materials by the researchers in the classroom. For five weekdays and two weekend days, all participants received the *MyMovez*^2^ *Wearable Lab*: A smartphone with a tailor-made research application and a wrist-worn accelerometer. The smartphone with the *MyMovez* application served as a measurement tool for the peer nomination and self-report items. Participants received daily questionnaires on these devices at random moments between 7:00 AM and 7:30 PM, except during school hours (i.e, they could receive questions during one of the school breaks).

### Measures

#### Physical activity

Physical activity was measured by a wearable accelerometer as number of steps per day. Wearable accelerometers are accurate and detailed instruments to measure physical activity [34]. The Fitbit Flex was used to measure physical activity, which has shown to be an accurate and reliable measurement of physical activity [35, 36]. Only complete measurement days were included, in which the accelerometer functioned the entire day and was worn by the participant. Therefore, measurements where only included if the total measured minutes equalled 1440 min (24 h), and at least 1000 steps were recorded. The first and the last day of the measurement period were partial days, because on these days the participants received or handed in the accelerometer. Therefore, the first and last day were excluded from the analyses. For analytical purposes, the steps per day variable was standardized across the remaining five days.

##### Missing data

In total, 73.37% of all possible data points were observed in the daily physical activity data (for a day-to-day overview, see Table 1). The Little’s MCAR test indicated that the data were not missing completely at random, χ^2^ (7) = 205.79, *p* < .001; relatively fewer data points were observed at the end of the week which was mostly caused by depleted batteries in the accelerometers. In addition, some participants had missing data for an entire week, caused by being absent at the start of the measurement period or a malfunction of the electronic devices (*n* = 18 at baseline, *n* = 28 during the intervention). Multilevel (predictive mean matching) imputation [37] was used to generate multiple imputations (100 imputations based on 500 iterations each) of the missing physical activity data. The missing data points were imputed based on other physical activity data of the participant, day of the week, measurement period, gender, age and athletic competence of the participant.

**Table 1:** Number (percentage) of valid data points for the physical activity data per day at baseline and intervention

	Day of measurement period				
Measurement period	Day 1	Day 2	Day 3	Day 4	Day 5
Baseline	172 (90.53%)	166 (87.37%)	159 (83.70%)	124 (65.26%)	108 (53.16%)
Intervention	162 (85.30%)	148 (77.90%)	147 (77.40%)	112 (58.90%)	103 (54.20%)

*N* = 1900

#### Sociometric nominations

Influence agents within each classroom were identified with the use of seven peer nomination questions. Three questions were based on the ASSIST-based studies: friendship, advise, and leadership [21]. The remaining four questions were based on peer influence mechanisms involving physical activity (i.e., “with whom do you hang out?”; “To whom do you want to come across as an active person?”; “Who does sports or activities that you also would like to do?”; and “With whom do you talk about physical activity?”) [38]. Participants could nominate peers of the same grade, by clicking on their names that were presented in a list on the research smartphone. Also, a search field was provided so participants could easily find the names of the friend that they want to select. Participants were free to nominate an unlimited number of peers but were required to nominate at least one other schoolmate (N.B. self-nominations were not possible).

##### Selection of influence agents

The most central participants were determined based on closeness centrality by entering all the sociometric nominations in the KeyPlayer package (version 1.0.3 [39]) in R (RStudio version 1.0.136, [40]). The KeyPlayer package uses a ‘greedy search algorithm’ to identify a specified number of influence agents that collectively represent the most central subgroup, adjusting for overlapping nominations within each classroom network [39]. This selection procedure differs from previous network interventions in which the researchers simply identified influence agents by selecting the participants that individually have the highest centrality without adjusting for redundant nominations. Additional analyses of the differences in selection criteria revealed that the overlap between influence agents identified using closeness centrality and those who would have been identified using the traditional criterion of indegree centrality was low (29%). This means that the influence agents selected in this study had a different position in the social networks compared to the agents identified in previous studies.

Based on previous research [19, 41], the top 15% of males and top 15% of females in each classroom were identified as influence agents. In total, 24 participants were identified as influence agents. Of the approached influence agents, 19 participants (42% male, age: 12–13 y/o) accepted the role, 1 participant declined, and 4 participants did not respond to the invitation. This resulted in four intervention classes including 4 influence agents, and the other intervention classroom including 3 influence agents.

#### Covariates

A number of covariates were included to adjust for possible confounding effects. Sex and age were included because males tend to be more active than females, and younger adolescents tend to be more active than older adolescents [42, 43]. In addition, athletic competence was measured by the *physical* subscale of the *self-perceived competence scale* [44]. This scale consisted of 10 items describing competence and interest in physical activity (e.g., “Are you good at sports?” or “Do you have confidence in doing new sports for the first time?”) that were measured on a 7-point likert scale (α = .84) ranging from “no, definitely not” (1) to “yes, definitely” (7).

### Social network intervention (SNI)

The training adapted elements from a training stimulating healthy drinking behavior used by Smit et al. [23]. The training was based on insights from the Self-Determination Theory (SDT) [45], targeting competence, autonomy and relatedness to increase adolescents’ motivation to be more physically active. In addition, the self-persuasion theory [46] was used to stimulate ownership of the targeted behavior. After the adaptation, all authors agreed on the face validity of the intervention. The intervention was pretested on two males and two females from the first grade of an unrelated secondary school. Based on their feedback adjustments were made, including the suggestion to refer to the influence agents as *team captains*.

The training consisted of four components: introduction, knowledge, skills and acceptance of the task. In the afternoon of the first day of the intervention, the influence agents received a message on their research phone that stated: “Based on the provided answers of the previous project week, you have been selected for a secret assignment. Together with a couple of classmates, you will carry out this assignment without the rest of the class knowing it”. Then, the role of the team captain (i.e., influence agent) was explained and questions about their own physical activity were asked to make the topic more salient. Subsequently, the training focused on knowledge about the benefits of physical activity. Based on self-persuasion theory [46], participants were first asked to name benefits of being physically active and the perception of their own physical activity. Next, the influence agents received eight benefits of physical activity (e.g., health, academic performance, enjoyment). To raise competence as an influence agent [45], the influence agents were thought influence strategies to promote physical activity in the classroom (based on [38, 46]). More specifically, the influence agents learned about four strategies: S*ocial facilitation* (by organizing an activity), *modelling* (by being an example and acting as a role model), *impression management* (by telling others about the benefits of physical activity and asking them why they are physically active), and *self-persuasion* (by asking others why they think physical activity is important). To increase their autonomy, the intervention emphasized that the team captains were free to use one or multiple influence strategies, and were also free to come up with other strategies. Lastly, the participants were asked to accept or decline the role of team captain. After accepting the role, the researchers contacted the team captains via the smartphone to reveal the identity of the other captains in the class, and check whether their role was clear. In the subsequent five days, all team captains received daily reminders on the benefits of physical activity and the four influence strategies.

#### SNI evaluation

After the intervention period, the influence agents filled out a questionnaire in which the intervention was evaluated. Specifically, the influence agents were asked about their role as a team captain, what types of strategies they used to performed their role and whether they thought they influenced the physical activity of the classmates.

### Strategy of analysis

In this study, five consecutive days of physical activity per participant were measured, resulting in a hierarchical data structure. Days of physical activity (level 1) were nested within participants (level 2), and participants were nested within school classes (level 3). Because of the hierarchical structure, random adjustments for the different levels to the fixed intercept were included [47]. For that reason, we used a linear mixed-effects model approach to account for the nested hierarchical structure of the physical activity data. The multilevel approach simultaneously controls for clustering of the data and gives more weight to participants with more days of physical activity data [48].

The data were cleaned, structured and analysed in R (RStudio version 1.0.136, [40]). Multilevel models were performed using the lme4 package [49]. Alpha was set at *p* < 0.05. First, the clustering of the data was assessed by examining the intraclass correlation coefficient (ICC) of the different levels. Adjustments per level were made in an additive manner where necessary. Subsequently, a randomization check was carried out to ensure that participants in each condition did not initially differ in physical activity. Then, the main analyses were performed by adding all parameters to the mixed models. The mixed models were performed on the data that included the imputed values as well as on the data that included only those with complete information to detect if the imputed data led to different results. Lastly, additional analyses were carried out to inspect the effect the intervention had on the influence agents.

## Results

### Preliminary analyses

#### Clustering of data

To examine the amount of variance in physical activity attributable to differences between classrooms, participants, and days of the week, three separate random-intercept models were performed and compared to the null model (only a fixed intercept). In each model, the standardized number of steps was included as the dependent variable. First, random intercepts per participants were added to the model specification. Likelihood ratio test indicated that the inclusion of random intercepts per classroom did not improve model fit, χ^2^(1) = .44, *p* = .51; but the inclusion of random intercepts per participant and per day did improve model fit (χ^2^(1) = 150.55, *p* < .001, and χ^2^(1) = 154.64, *p* < .001, respectively). That is, physical activity did not significantly vary between classrooms (ICC = .02), but did vary between participants (ICC = .24) and days (ICC = .12). Therefore, the subsequent models included random intercepts per participant and per day.

#### Randomization check

To test whether there were differences in physical activity at baseline between the conditions, a multilevel model was performed (that included random intercepts per participant and day). To check for possible differences between the influence agent and the non-influence agents in the social network intervention condition, the influence agents were treated as a separate condition. The control condition did not differ in physical activity from the influence agents, *b* = −.03, *SE* = .18, *p* = .85, or from the targeted adolescents, *b* = −.19, *SE* = .11, *p* = .09. This means that the randomization in terms of physical activity at baseline was successful. In addition, the participant characteristics in the different conditions were compared in sex, age, and athletic competence (Table 2). Adolescents were slightly older in the control condition, *F*(2, 187) = 8.69*, p* < .001. The difference in age between the conditions was accounted for in the subsequent analysis by including age as a covariate.

**Table 2:** Randomization checks of the covariates for the influence agents, SNI condition and control condition

	Condition			
	Influence agents	SNI	Control	*P* value^b^
Boys/girls (n/n)	8/11	32/42	48/49	.67
Age (y)	12.16 ± .37	12.00 ± .37	12.31 ± .57	<.001
Athletic Competence^a^	4.67 ± .82	4.68 ± .82	4.76 ± .75	.83

*N =* 190, ^a^Reflects the differences in means between the conditions by Pearson’s chi square test or one-way ANOVA. ^b^Likert scale [0–7]

### Main analyses

In the main analyses, we added the fixed effects of the measurement period (baseline vs. intervention), condition (SNI vs. control), the interaction between the measurement period and condition, and the covariates (sex, age and athletic competence) to the model with the random intercepts for days and participants.

**Fig. 2:**
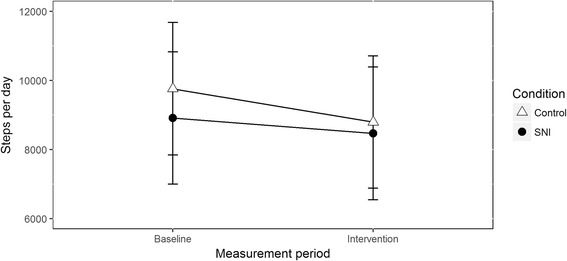
Estimated marginal mean steps per day (unstandardized) for the SNI condition and the control conditions at baseline and during the intervention week, after controlling for the clustering in data and all covariates. Unstandardized marginal means are presented for interpretation purposes

The final model included random intercepts per participant and day (sum-to-zero coded), fixed effects for the measurement period, condition, the interaction of measurement period and condition, sex, age and athletic competence. The number of steps was significantly predicted by measurement period (*b* = − 0.18, *SE* = .08, *p* = .033). In both conditions, participants were more active at baseline (*M* = 9334.23, *SD* = 771.39 steps per day) than during the intervention week (*M =* 8629.00, *SD =* 772.16 steps per day). The number of steps was not predicted by the condition (*b* = 0.14, *SE* = .10, *p* = .151). Also, we did not observe a statistically significant interaction between the measurement period and condition in the data with imputed values (*b* = .04, *SE* = .10, *p* = .66) nor in the data without imputed values (*b* = .10, *SE = .09*, *p* = .27). This means that changes in physical activity between baseline and intervention did not differ between the SNI group and the control group (see Fig. 2). Contrary to the hypothesis that adolescents in the SNI-condition would increase their physical activity over time compared to the control condition, no effect of the intervention was observed.

With regard to the covariates, the number of steps taken was significantly predicted by sex (*b* = − 0.39, *SE =* .09, *p* = .001). On average, boys (*M =* 9988.48, *SD* = 798.52 steps per day) were more active than girls (*M =* 7974.75, *SD* = 778.47 steps per day). Likewise, athletic competence predicted the number of steps per day (*b* = 0.18, *SE = .*04, *p* < .001). Adolescents who were more athletically competent were more physically active. As can be seen in Table 3 (Table 4 for the complete case analysis), age did not affect physical activity.

**Table 3:** Linear mixed-effects model for the standardized physical activity for the imputed dataset

			s^2^	*b*	*SE*	DF	*p*	95% CI
Random	Participant	Intercept	0.16					
	Day	Intercept	0.13					
		Residual	0.62					
Fixed		Intercept		.75	1.09	411.47	.49	[−1.39, 2.89]
		M. period		−.18	.08	39.243	.033*	[−.34, −.01]
		Condition		−.14	.10	426.30	.151	[−.33, −.05]
		Sex		−.39	.09	223.25	<.001*	[−.56, −.21]
		Age		−.04	.09	456.15	.67	[−.21, .14]
		Athletic competence		.18	.04	455.43	<.001*	[.09, .26]
		M. Period * Condition		.04	.10	85.05	.66	[−.15, .24]

Note. *N* = 190. CI = Confidence interval. Marginal R^2^ = 0.07. Conditional R^2^ is not applicable for multiple imputed mixed models. *Significant at *p* < 0.05

**Table 4:** Linear mixed-effects model for the standardized physical activity for the complete cases dataset

			s^2^	*b*	*SE*	DF	*p*	95% CI	
Random	Participant	Intercept	0.16						
	Day		Intercept	0.13					
		Residual	0.62						
Fixed		Intercept		.73	1.03	157.30	.48	[−1.28, 2.74]	
		M. period		−.19	.07	1159.50	.004*	[−.32, −.06]	
		Condition		−.17	.09	245.60	.07	[−.35, .01]	
		Sex		−.40	.08	152.50	.001*	[−.56, −.24]	
		Age		−.03	.08	153.70	.70	[−.19, .13]	
		Athletic competence		.18	.03	149.40	<.001*	[.10, .26]	
		M. Period * Condition		.10	.09	1152.60	.27	[−.08, .28]	

Note. *N* = 190. CI = Confidence interval. Marginal R^2^ = 0.09. Conditional R^2^ = 0.39. *Significant at p < 0.05

#### Influence agents’ evaluation

After the intervention measurement period, the 19 influence agents received a post-intervention evaluation (Table 5) to which 57.9% responded. The qualitative data show that most of the influence agents indicated that they were neutral to positive about being an influence agent and thought it was an easy task to perform, while some others did not like the role or thought it was hard to promote physical activity. Additionally, most influence agents indicated that they were not aware of their influence on others and not sure if they had increased physical activity among their classmates. The influence agents indicated that all the different tactics from the training to promote physical activity were used, with modelling being the most popular. The influence agents performed their task throughout the day, so not only during school hours. Lastly, almost all influence agents indicated that they did not use social media to perform their task.

**Table 5:** Responses to the Evaluation of the SNI by the influence agents

	*M*	*SD*
How did you like being a team captain?		
0 = not at all, 100 = very much	62.82	25.00
How hard was the task of being a team captain?		
0 = very easy, 100 very hard	42.73	31.37
	%	
Which tactics did you use to promote physical activity		
Impression management	25.00	
Modelling	41.67	
Social facilitation	16.67	
Self-persuasion	16.67	
At what time during the day did you carry out the role the most		
Before school	12.50	
During the breaks	25.00	
During class	31.25	
After school	31.25	
Did you use social media for your role?		
Yes	9.09	
No	90.91	
Do you think you were successful in increasing the physical activity of classmates?		
Yes	27.27	
No	9.09	
Don’t know	63.64	

*N* = 11

## Discussion

This study was one of the first to test the effectiveness of a social network intervention to promote physical activity among adolescents. In addition, the study selected the influence agents based on their closeness centrality within the social networks, and used an innovative approach to train the influence agents via smartphones. Contrary to our expectation, we did not find an effect of the social network intervention on the physical activity of adolescents.

The findings are not in line with previous social network interventions promoting other types of health behaviors than physical activity [20, 21, 23, 24]. In these studies, social network interventions have shown promising results to promote a variety of health behaviors. When focusing on physical activity, our findings are not in line with Sebire et al. [27] who was successful in promoting physical activity in adolescent females via a social network intervention. However, our study shows similar results as Bell et al. [31], who observed no social network intervention effect on dietary intake or physical activity. Their main recommendation was that the training should be relatively simple and the intervention message should be easy to pass on. Bell et al. advised focussing the intervention on one health behavior at a time. Our study followed this advice and focused only on physical activity. However, this did not increase the effectiveness of the social network intervention. In our view, there are two plausible explanations for the discrepancy between our study and the previously discussed social network interventions.

One explanation for our finding is that we adjusted the existing social network interventions to a smartphone environment to increase feasibility and make the intervention more fun and suitable for large-scale deployment. This study was the first to incorporate smartphones in a social network intervention. Influence agents were approached and trained via the research app in the *Wearable Lab*. This is a less personal approach compared to previous social network intervention studies in which the influence agents met face-to-face with their trainers and other influence agents [20, 21, 23, 31]. Also, because of the smartphone-based training, the instructions took less time compared to previous studies. It might have resulted in less commitment and team effort to perform their task. Although the influence agents indicated at the end of the intervention that they liked their role, it was unclear whether they completely understood the training and were motivated to be influence agents. In order to decrease the psychological distance between the researchers and the influence agents, we added a photograph of the researcher who gave the instruction in the school to the training and contacted the influence agents personally via the smartphone after completing the training. A more personal approach has been successfully used by Smith et al. [50] in a smartphone obesity prevention trial to promote physical activity for boys with an increased risk of obesity. Apart from three interactive seminars at school focusing on increasing physical activity and decreasing screen-time, participants used a smartphone application to receive feedback and to keep in touch with the researchers. Future research could adapt this to social network interventions by combination between personal contact (e.g., at the start of or during the intervention) and contact via the smartphone (e.g., during the intervention), and test whether this approach is a feasible tool for training and a way to keep in contact with the influence agents.

Another explanation for our findings involves our approach to use closeness centrality as a means of identifying the influence agents. Previous social network interventions have exclusively used indegree centrality to identify influence agents [20, 21, 23, 27, 31]. Based on the idea that individuals who receive the most nominations would be reluctant to change behavior because they want to remain popular [28], we opted to use closeness centrality because these individuals were expected to have more influence within the entire network when it comes to the promotion of health behavior [30]. A possible consequence is that the influence agents in our study were closely connected to all the other classmates, but were not effective in persuading others because they did not have a high status. Future research should further investigate the selection of the influence agents by systematically evaluating the effectiveness of influence agents identified by these (and other) selection criteria. By doing so, the generalizability of the diffusion mechanism of the health campaign will become more clear.

### Limitations

Innovative studies go along with a number of limitations and several limitations should be discussed in interpreting the results. First, active parental consent was required for participants to be included in this study due to ethical and legal considerations. As a result, there were some students in each classroom that did not participate, which may have influenced the identification of the influence agents in the social network. That is, the adolescents who did not participate did not provide nominations nor could they be nominated by participants. It also remains unclear whether non-participants differed in their physical activity compared to the participants. It could be that the non-participants did not want to participate because of their sedentary lifestyle. To reduce this potential confound, however, classes with a high percentage of non-participants (participation lower than 60%) were excluded.

Second, only one large school was approached to participate in order to reduce potential differences between the classes in the control condition and in the intervention condition. This may have had an effect on the external validity. Future research should include multiple schools to examine whether differences occur between different locations or school types, and make the results more generalizable.

Third, compared to other social network studies, the intervention period was rather short. In previous studies, the intervention period lasted for multiple weeks. A longer intervention period enables more opportunities for the influence agents to perform their role and influence the behavior of the rest of the class. Due to time constraints of the participating school and limited availability of the research material, the intervention period in this study was only one week. Future research should consider using a longer intervention period than a week to provide more time for the influence agents to promote the health behavior among their classmates.

## Conclusion

Despite these limitations, this study advanced the field of social network interventions in three ways. First, the present study was the first social network intervention that used a ‘greedy search algorithm’ to identify influence agents based on closeness centrality. Although we did not directly compare influence agents identified using a different criterion, our study extended social network theory by using an alternative selection criterion that reflects the main tenets of social network theory. Our study provides implications for future research to build on this extended way of thinking about the role of influence agents.

Second, this study was the first that used smartphones to train the influence agents in a social network intervention to promote physical activity in adolescents. Evaluations showed that research using smartphones is a feasible research tool to not only collect various types of data, but also to train and keep in touch with the influence agents. Nevertheless, maintaining personal contact with influence agents is still an important aspect to consider.

Third, the present study used a sophisticated analytic procedure utilizing multilevel analyses and multiple imputations, to adjust for the nested structure of the data and to include individuals with missing values. This procedure provided a more stringent test of the intervention effect by accounting for variance in physical activity due to daily fluctuations in activity levels and to individual differences.

In this study, we did not observe an effect of the social network intervention on the physical activity of adolescents. However, given that social network interventions in physical activity (as well as other health behaviors) are relatively underutilized and understudied, we encourage continued research applying social network interventions among adolescents to promote health behaviors and advance behavioral health science.

## Abbreviations

ECSW: Ethiek Commissie Sociale Wetenschappen (Ethics Committee Social Sciences)
ERC: European Research Council
HAVO/VWO: Hoger Algemeen Voortgezet Onderwijs/Voorbereidend Wetenschappelijk Onderwijs (theoretical training)
ICC: Intraclass correlation coefficient
MVPA: moderate-to-vigorous physical activity
NTR: Nederlands Trial Register (Dutch Trial Registry)
SDT: Self-Determination Theory
SNI: Social Network Intervention
VMBO: Voortgezet middelbaar beroepsonderwijs (vocational training)

## Footnotes

1 The design was adjusted compared to the original protocol design (i.e., two conditions were omitted because lower participation due to active consent procedures).

2 For more information about the project see: www.mymovez.eu.
